# The effect of *Broussonetia papyrifera* silage on intestinal health indicators and fecal bacterial composition in Kazakh sheep

**DOI:** 10.3389/fvets.2025.1543302

**Published:** 2025-02-26

**Authors:** Xiaokai Zheng, Yingchao Sun, Sijin Guo, Junyang Yu, Rongzheng Huang, Fanfan Zhang

**Affiliations:** College of Animal Science and Technology, Shihezi University, Shihezi, China

**Keywords:** *Broussonetia papyrifera*, antibiotic-free feed, immunity, antioxidation, fecal bacteria

## Abstract

Hybrid *Broussonetia papyrifera* shows great promise for use in antibiotic-free feed, potentially contributing to the green and sustainable development of the animal husbandry industry. In this study, we investigated the impact of *Broussonetia papyrifera* silage on the intestinal health of Kazakh sheep. Forty healthy male Kazakh sheep, aged 5 months and weighing an average of 28.28 ± 1.14 kg, were randomly assigned to either a control or an experimental group, each comprising four replicates, with five sheep per replicate. The control group was fed a basal diet, while the experimental group received a diet supplemented with 20% *Broussonetia papyrifera* silage (dry matter basis). The 70-day experiment included a 10-day adaptation phase followed by a 60-day feeding trial. The results showed that there was no significant difference in growth performance or apparent nutrient digestibility between the experimental and control groups (*p* > 0.05). However, the experimental group exhibited significantly greater total antioxidant capacity, alongside higher contents of superoxide dismutase, catalase, glutathione peroxidase, immunoglobulins A, M, and G, and interleukins-2, −6, and −8 in the intestinal mucosa; in contrast, malondialdehyde and interleukin-4 contents were significantly reduced (*p* < 0.01). Furthermore, the dietary inclusion of *Broussonetia papyrifera* silage resulted in a reduction in the relative abundance of the bacterial genera *Turicibacter* and *Romboutsia* (*p* < 0.05). In conclusion, the feeding of *Broussonetia papyrifera* silage to Kazakh sheep significantly enhanced immune function, increased antioxidant capacity, and reduced the relative abundance of potentially pathogenic bacteria in the sheep without negatively impacting their growth or nutrient digestion, thus supporting the overall health of the animals.

## 1 Introduction

Livestock husbandry represents a critical source of meat for humans. The use of antibiotics in livestock feed has been banned in many countries to mitigate the risk of animal-derived antibiotic resistance, thereby safeguarding human health (1). Accordingly, research interest has increasingly focused on the development of alternative, antibiotic-free feed additives that can enhance nonspecific immunity in animals (2, 3). Additionally, the advancement of “green, efficient, and safe” feeds, free not only from antibiotics but also from hormones and exogenous chemical agents, represents a significant trend for the future development of the feed industry.

*Broussonetia papyrifera*, a cultivar recently developed by the Institute of Botany at the Chinese Academy of Sciences, is rich in diverse bioactive compounds such as flavonoids, polysaccharides, and terpenoids (4). It was recently shown that *B. papyrifera* ensilage treatment enhances its assimilation and absorption by animals (5). The silage derived from *B. papyrifera* holds significant potential for applications related to animal health and is being explored as a potential alternative to antibiotics (6–11). *B. papyrifera* silage has been shown to benefit animal health by improving growth performance, immune function, and antioxidant capacity, as well as through its modulatory effects on ruminal bacterial communities (12–18). While numerous studies have investigated the effects of *B. papyrifera* silage on ruminal bacteria, few have addressed its impact on fecal bacteria in animals. Bacteria in feces reflect the broader microbial ecosystem of the digestive tract of ruminants and offer insights into the effects of diet, digestive efficiency, microbial health, and immune status (19). *B. papyrifera* silage has shown different effects on the health of different animals such as sheep, goats, rabbits, cows, donkeys, and piglets (12–16). In summary, incorporating *Broussonetia papyrifera* silage into livestock diets can reduce reliance on antibiotics and exogenous chemicals, thereby promoting more sustainable green development by enhancing feed efficiency and minimizing environmental pollution in agricultural cycles.

Kazakh sheep, originating in China, are highly valued for their ability to endure harsh environments, a quality that is particularly beneficial for nomadic herders in arid and semi-arid regions. To the best of our knowledge, research on the effects of *B. papyrifera* silage on the health of Kazakh sheep is limited. Therefore, in this study, we explored the effects of *B. papyrifera* silage on growth performance, intestinal health, and fecal bacterial composition in these animals, aiming to provide a scientific foundation for its application in green, antibiotic-free ruminant breeding.

## 2 Materials and methods

The animal research protocol used in this study was approved by the biological ethics committee of Shihezi University (Shihezi, China) in March 2023, under approval number A2023-129.

### 2.1 Preparation of silage from *Broussonetia papyrifera*

The experimental site was located at the *B. papyrifera* demonstration base of the Seventh Agricultural Science Institute in Xinjiang Province (N 44°20′, E 83°51′, elevation 450 m). *B. papyrifera* was harvested on April 20, 2023, at a height of 120 cm, leaving a stubble of approximately 10 cm, and the entire plant was cut into 2–3 cm-long pieces. The plants were then inoculated with 1 × 10^5^ CFU/g *Lactiplantibacillus plantarum* (isolated from *B. papyrifera* silage) and thoroughly mixed (4). The *B. papyrifera* silage was then wrapped and sealed, with each package weighing approximately 80 kg. The silage underwent fermentation for 60 days at a storage temperature of 24°C. Nutrient analysis was performed on the dry matter of *Broussonetia papyrifera* silage after fermentation, and the results are presented in Table 1.

**Table 1:** Nutritional levels of *Broussonetia papyrifera* silage (dry matter basis).^1^

Item	Content	SEM
pH	4.58	0.004
Dry matter	35.11	0.102
Crude protein	17.88	0.101
Neutral detergent fiber	42.78	0.303
Acid detergent fiber	30.16	0.635
Water-soluble carbohydrate	8.32	0.106
Crude ash	10.42	0.069

^1n = 4; SEM, standard error of the mean.^

### 2.2 Animal experimentation and experimental design

A total of 40 male Kazakh sheep (castrated rams), approximately 5 months old and weighing an average of 28.28 ± 1.14 kg, were selected for the study. The sheep were randomly divided into a control group (CK) and an experimental group (GS), each with four replicates (five sheep per replicate). The control group was fed a basal diet, while the experimental group received a total mixed ration containing *B. papyrifera* silage (20% dry matter). The formulation of the experimental diet followed the Chinese Sheep Feeding Standard (NY/T 816–2021) and was designed to meet the nutritional requirements for the growth phase of sheep. Detailed information on the composition and nutrient levels of the experimental diet is provided in Table 2. The crude-to-refined ingredient ratio was 4:6. The feeding period lasted for 10 days, followed by a 60-day trial period. The experiment was conducted in Jinghe County, Xinjiang Province (N 82°30′49.20″, E 44°31′19.58″, elevation 384 m). Each sheep was individually housed in pens and had ad libitum access to water throughout the study. The sheep were fed twice daily at 08:00 and 18:00 h, with feed leftovers restricted to approximately 5%.

**Table 2:** Composition and nutrient levels of the experimental diets (dry matter basis).

Item	Control group	Experimental group
Ingredients		
Corn (%)	35.00	35.00
Wheat straw (%)	15.00	15.00
Soybean meal (%)	6.00	6.00
Cottonseed meal (%)	3.00	3.00
Alfalfa hay (%)	15.00	12.00
Total corn silage (%)	22.00	5.00
*Broussonetia papyrifera* silage (%)	-	20.00
NaCl (%)	0.30	0.30
Baking soda (%)	0.70	0.70
Premix^1^	3.00	3.00
Total	100.00	100.00
Nutrient levels		
Metabolic energy (MJ/kg)	11.15	11.19
Crude protein (%)	14.12	13.92
Neutral detergent fiber (%)	35.55	35.81
Acid detergent fiber (%)	26.30	26.36
Calcium (%)	0.64	0.63
Phosphorus (%)	0.52	0.53

^1Each kg of premix contained the following: vitamin A, 3,000 IU; vitamin D, 7,500 IU; vitamin E, 19 IU; Cu, 18.5 mg; Zn, 118.5 mg; Fe, 81 mg; Mn, 118.5 mg; Se, 0.75 mg; and Co, 1.75 mg. Metabolic energy was a calculated value, while the others were measured values.^

### 2.3 Sample collection

On the final day of the experimental period, five sheep from each group with body weights closest to the average weight of the group were randomly selected for fecal sample collection. Samples were obtained from the sheep 4 h after the morning feeding. Rectal fecal samples (approximately 0.2 g each) were stored in a liquid nitrogen tank for microbial community analysis. Subsequently, 3 sheep from each group (6 sheep in total) were randomly selected from those that provided fecal samples for slaughter. After slaughter, the abdominal cavity was quickly opened, and the middle sections of the duodenum, jejunum, and ileum were extracted and stored at −80°C for subsequent analysis (20).

### 2.4 Measurement of indicators

#### 2.4.1 Feed nutrient levels

Dry matter content was determined according to the AOAC standard procedure. Calcium (Ca) content was determined using AOAC official method 968.08, phosphorus (P) content was determined using AOAC official method 965.17, acid detergent fiber (ADF) content was determined using AOAC official method 973.18, the nitrogen content of the feed was measured using the Kjeldahl method, and the neutral detergent fiber (NDF) content was determined according to Van Soest (21).

#### 2.4.2 Growth

On days 1 and 60 of the experiment, the body weight of the sheep was measured in the morning after an overnight fast.

#### 2.4.3 Apparent nutrient digestibility

On day 53 of the experiment, five sheep were randomly selected from each group, and fecal samples were continuously collected from these sheep for 7 days using the total feces collection method. The collected feces were thoroughly mixed, and 10% of the sample was treated with 10% sulfuric acid for nitrogen preservation. The samples were then dried at 65°C and stored for subsequent determination of fecal nutrient contents. During this period, feed and leftover feed samples were also collected, and their nutrient content was determined using the method outlined in Section 2.4.1. The apparent digestibility of nutrients in the experimental animals was calculated based on the acid-insoluble ash (AIA) content (21).

#### 2.4.4 Determination of antioxidant, immunoglobulin, and cytokine contents

Duodenal, jejunal, and ileal mucosal tissues were individually weighed to 0.1 g. Subsequently, 0.9 mL of prechilled 0.9% physiological saline was added to each sample, and the mixture was homogenized using a homogenizer to obtain a 10% tissue homogenate. A 0.5-mL aliquot of the resulting homogenate was then centrifuged at 2000 rpm for 10 min at 4°C and the resulting supernatant was stored at −20°C for subsequent analysis (22). Intestinal tissue indices in the supernatants were analyzed using commercial kits (Shanghai Meilian Biological Technology Co., Ltd., Shanghai, China) according to the manufacturer’s instructions. The antioxidant indices measured were total antioxidant capacity (T-AOC) and superoxide dismutase (SOD), catalase (CAT), glutathione peroxidase (GSH-Px), and malondialdehyde (MDA) contents. The immunoglobulins assessed were immunoglobulin (IgA), IgG, and IgM. The cytokines evaluated included tumor necrosis factor-alpha (TNF-*α*), interleukin-2 (IL-2), IL-4, IL-6, and IL-8.

#### 2.4.5 Fecal bacteria

The collected (cryogenically frozen) fecal samples were placed in 200 mL of sterilized triangular flasks, mixed with 50 mL of PBS (pH 7.2), shaken at 200 rpm for 30 min, ultrasonicated at 50 W for 2 min, and subsequently shaken at 150 rpm for another 30 min. The supernatant was then transferred to 50-mL sterilized centrifuge tubes that had been sterilized under high pressure and centrifuged at 1,500 rpm for 1 min. After transferring to new 50-mL sterilized high-speed centrifuge tubes, the samples were centrifuged at 12,000 rpm for 10 min, and the isolated bacteria were collected. DNA was extracted from the fecal samples using a DNA extraction kit (QIAamp PowerFecal DNA Kit, Qiagen, Hilden, Germany) and assessed for concentration and purity using 1% agarose gel electrophoresis.

The V4–V5 region of the bacterial 16S rRNA gene was amplified using the primers 515F (5′-GTGYCAGCMGCCGCGGTAA-3′) and 926R (5′-CCGYCAATTYMTTTRAGT-3′). PCR amplification was performed in 20-μL volumes using the following parameters: 95°C for 3 min, followed by 30 cycles of 94°C for 20 s, 55°C for 20 s, and 72°C for 30 s, with a final extension at 72°C for 10 min. Amplicons were verified by agarose gel electrophoresis, purified, and sent to Meij Biotech for library construction. Sequencing was performed on the Illumina MiSeq platform using paired-end reads (300 bp). Raw reads underwent quality filtering with fastp (v0.19.6) and merging with FLASH (v1.2.11). Denoising was performed using the DADA2 plugin in QIIME2 (default parameters). Denoised sequences (ASVs) were rarefied to 20,000 sequences per sample, yielding an average Good’s coverage of 99.09%. Taxonomic classification was conducted using the Naive Bayes classifier in QIIME2 with the SILVA 16S rRNA database (version 138). Subsequent analyses were performed on the Majorbio Cloud Platform.^1^ The original data relating to fecal bacteria obtained in this study can be accessed at https://www.ncbi.nlm.nih.gov/sra/PRJNA1167466, with SRA accession number PRJNA1167466.

### 2.5 Statistical analysis

Data were preprocessed using Excel 2018 and analyzed with SPSS 20.0. Prior to applying independent samples t-tests or one-way ANOVA, data normality was assessed using the Shapiro–Wilk test and homogeneity of variances with Levene’s test. Non-parametric tests were applied if assumptions were not met. Post-hoc comparisons were performed using Tukey’s test (*p* < 0.05). The classification and abundance of bacteria in the fecal samples were analyzed using the Majorbio Cloud Platform. All of data analysis in the biological cloud platform (see Footnote 1), specific as follows: Alpha is obtained by using the mothur software^2^ diversity sobs, chao, shannon index, and USES the Wilxocon rank-sum test for Alpha diversity analysis of differences between groups; The similarity of microbial community structure among samples was tested by PCoA analysis (principal coordinate analysis) based on bray-curtis distance algorithm, and the PERMANOVA non-parametric test was used to analyze whether the difference in microbial community structure between sample groups was significant. Species were selected for correlation network analysis based on spearman correlation |r| > 0.6 and *p* < 0.05.

## 3 Results

### 3.1 Growth performance and the apparent digestibility of nutrients

As shown in Table 3, no significant difference in growth performance or apparent nutrient digestibility was detected between the GS and CK groups (*p* > 0.05).

**Table 3:** The effect of *Broussonetia papyrifera* silage on the growth performance of Kazakh sheep.

Item	Group		SEM	*P*-value
	CK	GS		
Initial weight (kg)	28.26	28.28	0.036	0.859
Final weight (kg)	35.53	35.72	0.015	0.746
Apparent digestibility of dry matter (%)	64.38	63.74	2.521	0.820
Apparent digestibility of crude protein (%)	69.15	68.75	2.156	0.753
Apparent digestibility of crude lipid (%)	83.32	84.31	1.173	0.720
Apparent digestibility of neutral detergent fiber (%)	56.17	53.47	2.906	0.627
Apparent digestibility of acid detergent fiber (%)	45.38	44.99	2.543	0.789

SEM, standard error of the mean. Differences were considered significant at *p* < 0.05.

### 3.2 Antioxidant capacity

As shown in Table 4, T-AOC and the levels of SOD, CAT, and GSH-Px in duodenal, jejunal, and ileal tissues in the GS group were significantly elevated compared to those in the CK group (*p* < 0.01), whereas the MDA content was significantly reduced (*p* < 0.01).

**Table 4:** The effect of *Broussonetia papyrifera* silage on the antioxidant capacity of intestinal tissues in Kazakh sheep.

Item	Group		SEM	*P*-value
	CK	GS		
Duodenal mucosa				
T-AOC (pg/mL)	53.82	149.87	19.702	< 0.01
SOD (pg/mL)	70.31	165.57	19.771	< 0.01
CAT (pg/mL)	318.58	715.37	81.032	< 0.01
GSH-Px (pg/L)	217.34	472.67	53.622	< 0.01
MDA (nmol/L)	6.47	2.94	0.741	< 0.01
Jejunal mucosa				
T-AOC (pg/mL)	81.03	156.97	15.897	< 0.01
SOD (pg/mL)	83.49	163.11	16.225	< 0.01
CAT (pg/mL)	337.50	734.30	82.546	< 0.01
GSH-Px (pg/L)	233.61	468.87	48.794	< 0.01
MDA (nmol/L)	5.72	2.88	0.602	< 0.01
Ileal mucosa				
T-AOC (pg/mL)	67.17	153.94	17.827	< 0.01
SOD (pg/mL)	71.69	168.59	19.998	< 0.01
CAT (pg/mL)	393.65	715.37	67.836	< 0.01
GSH-Px (pg/L)	214.12	479.27	54.418	< 0.01
MDA (nmol/L)	6.22	2.94	0.687	< 0.01

SEM, standard error of the mean. Differences were considered significant at *p* < 0.05. T-AOC, total antioxidant capacity; SOD, superoxide dismutase; CAT, catalase; GSH-Px, glutathione peroxidase; MDA, malondialdehyde.

### 3.3 Immunoglobulin concentrations

As shown in Table 5, the contents of IgA, IgM, and IgG in the duodenal, jejunal, and ileal tissues were all significantly higher in the GS group than in the CK group (*p* < 0.01).

**Table 5:** The effect of *Broussonetia papyrifera* silage on the immunoglobulin content in the intestinal tissues of Kazakh sheep.

Item	Group		SEM	*P*-value
	CK	GS		
Duodenum mucosa				
IgA (μg/mL)	119.85	258.20	29.482	< 0.01
IgM (μg/mL)	3.77	9.49	1.262	< 0.01
IgG (μg/mL)	320.26	802.22	99.066	< 0.01
Jejunal mucosa				
IgA (μg/mL)	111.09	236.18	25.854	< 0.01
IgM (μg/mL)	3.67	9.64	1.303	< 0.01
IgG (μg/mL)	347.73	782.98	89.545	< 0.01
Ileal mucosa				
IgA (μg/mL)	113.00	227.74	23.704	< 0.01
IgM (μg/mL)	2.97	10.11	1.498	< 0.01
IgG (μg/mL)	342.41	881.89	114.105	< 0.01

SEM, standard error of the mean. Differences were considered significant at *p* < 0.05. IgA, immunoglobulin A; IgG, immunoglobulin G; IgM, immunoglobulin M.

### 3.4 Cytokine contents

As listed in Table 6, the levels of TNF-*α*, IL-2, IL-6, and IL-8 in the duodenal, jejunal, and ileal tissues of the GS group were significantly higher than those in the CK group (*p* < 0.01), with only IL-4 contents being significantly lower in the GS group than in the control group (*p* < 0.01).

**Table 6:** The effect of *Broussonetia papyrifera* silage on the cytokine contents of intestinal tissue in Kazakh sheep.

Item	Group		SEM	*P*-value
	CK	GS		
Duodenal mucosa				
TNF-α (ng/L)	282.81	773.59	99.463	< 0.01
IL-2 (pg/mL)	377.09	867.25	100.765	< 0.01
IL-4 (pg/mL)	94.22	45.07	10.564	< 0.01
IL-6 (pg/mL)	66.35	171.96	22.147	< 0.01
IL-8 (pg/mL)	14.43	38.69	5.028	< 0.01
Jejunal mucosa				
TNF-α (ng/L)	278.77	753.70	96.795	< 0.01
IL-2 (pg/mL)	407.98	935.40	109.476	< 0.01
IL-4 (pg/mL)	95.58	52.43	9.124	< 0.01
IL-6 (pg/mL)	58.69	169.47	22.925	< 0.01
IL-8 (pg/mL)	15.05	41.25	5.407	< 0.01
Ileal mucosa				
TNF-α (ng/L)	275.12	724.87	93.628	< 0.01
IL-2 (pg/mL)	354.27	966.30	128.594	< 0.01
IL-4 (pg/mL)	92.79	50.71	8.802	< 0.01
IL-6 (pg/mL)	67.71	168.88	21.475	< 0.01
IL-8 (pg/mL)	14.59	37.93	4.767	< 0.01

SEM, standard error of the mean. Differences were considered significant at *p* < 0.05. TNF-α, tumor necrosis factor-alpha; IL, interleukin.

### 3.5 Fecal bacteria

**Figure 1:**
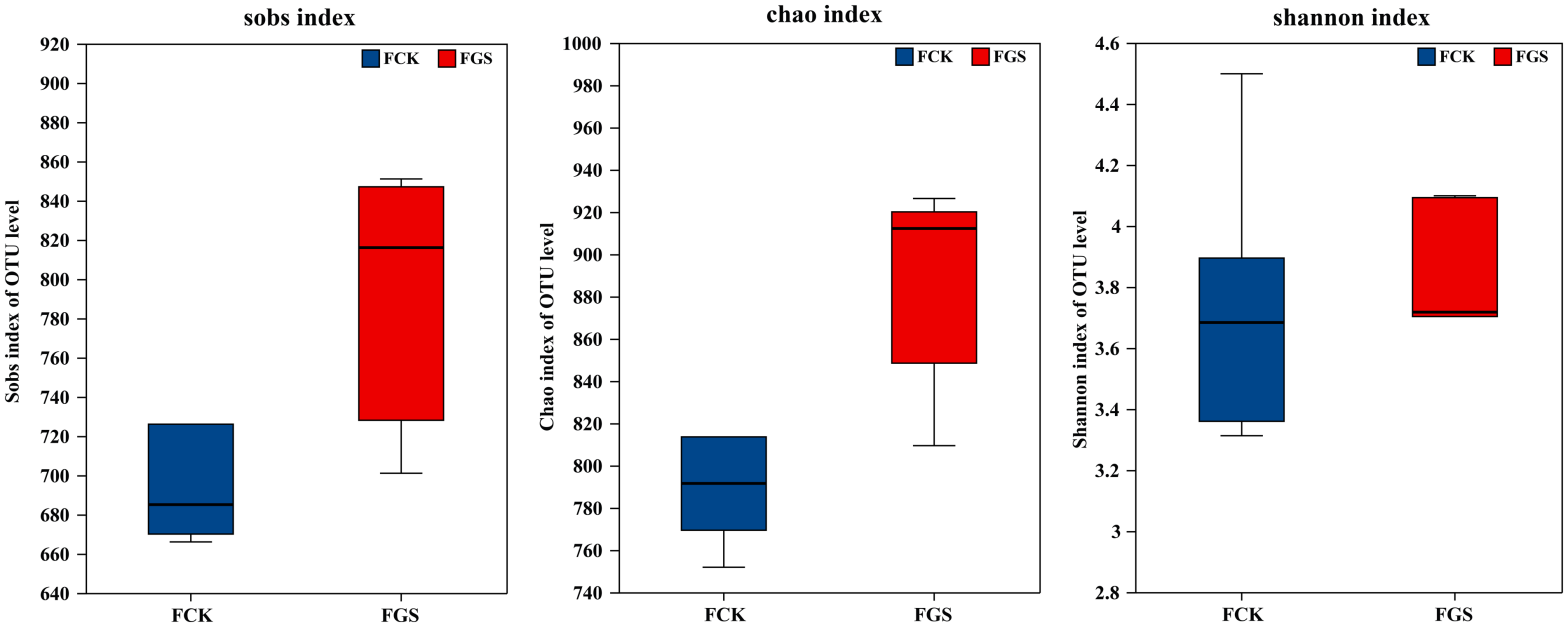
Box plots of alpha diversity analysis. FCK, fecal bacterial samples collected from the control group; FGS, fecal bacterial samples collected from the *Broussonetia papyrifera* silage treatment group; OTU, operational taxonomic unit.

As shown in Figure 1, the Sobs, Chao, and Shannon indices were all higher in the GS group than in the CK group; however, the differences were not significant (*p* > 0.05).

**Figure 2:**
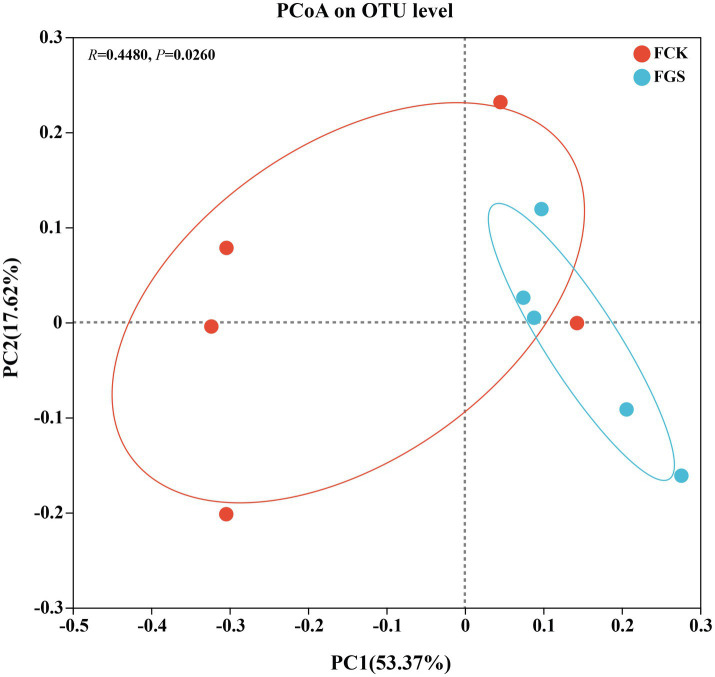
Principal coordinate analysis (PCoA). The horizontal and vertical coordinates represent the two selected principal coordinate components. The percentage represents the contribution of each principal coordinate component to the difference in sample composition. The closer the *R*-value is to 1, the greater the between-group difference relative to the within-group difference; conversely, the smaller the *R*-value is, the less significant the between- and within-group differences.

Principal coordinate analysis was used for the evaluation of beta diversity among fecal microbes (Figure 2). The results showed that microbial composition differed significantly between the treatment groups (*R* = 0.4480, *p* < 0.05). The R-value indicates the correlation between sample groups, with higher R-values indicating greater dissimilarity between groups.

**Figure 3:**
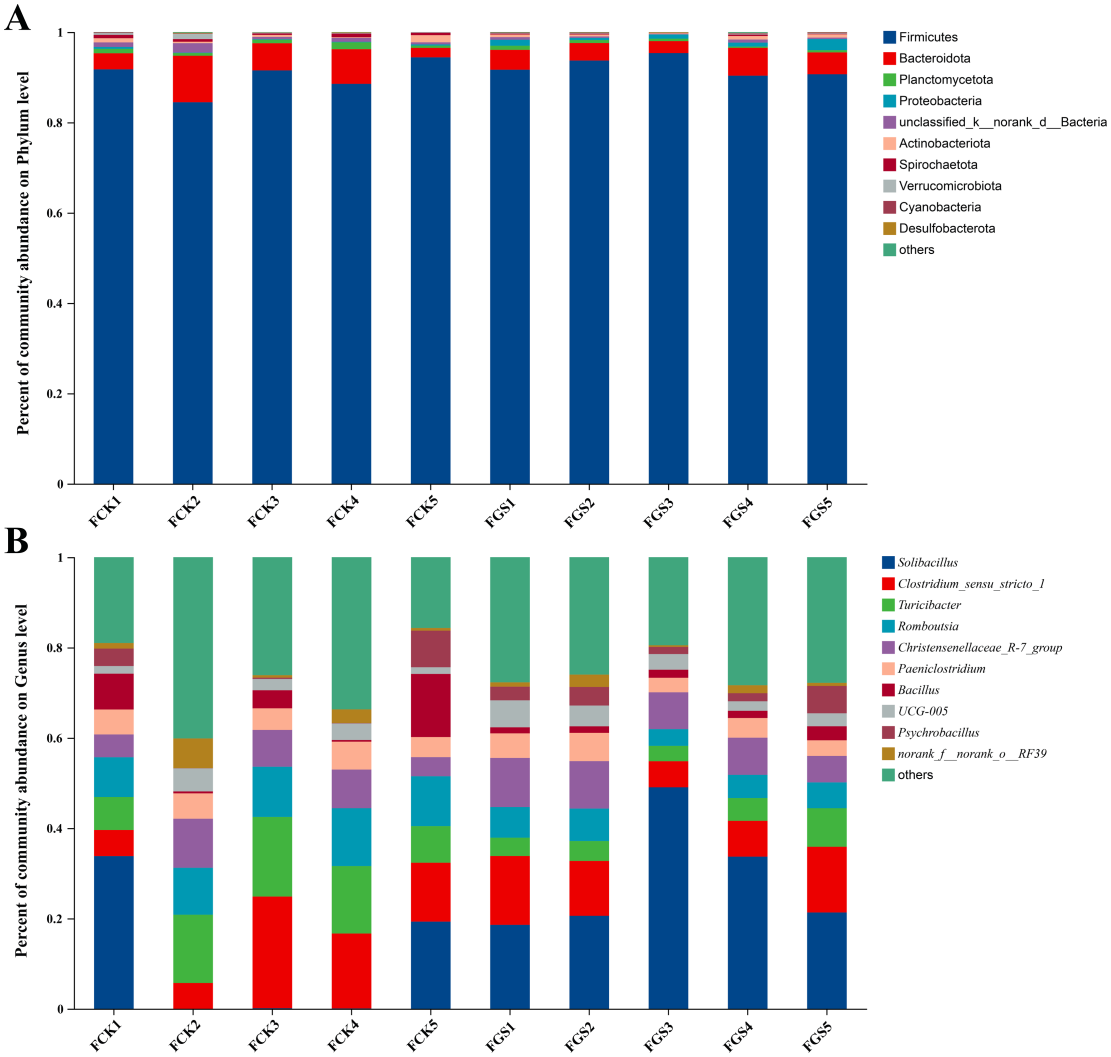
Fecal bacterial communities at the phylum **(A)** and genus **(B)** levels. FCK, fecal bacterial samples collected from the control group; FGS, fecal bacterial samples collected from the *Broussonetia papyrifera* silage treatment group.

As shown in Figure 3A, *Firmicutes* was the dominant phylum in fecal bacterial samples collected from both the control (FCK) and *Broussonetia papyrifera* silage treatment (FGS) groups, with relative abundances of 86.34–93.92% and 90.21–94.47%, respectively, followed by *Bacteroidota*, with relative abundances of 2.58–9.28% and 3.10–5.68%, respectively.

At the genus level, *Solibacillus* was the dominant bacterial genus in both the FCK and FGS groups (relative abundances: 15.39–26.05 and 15.78% ~ 41.50%, respectively), followed by *Clostridium*_sensu_stricto_1 (relative abundances: 5.17% ~ 21.17 and 7.00% ~ 15.28%, respectively), *Turicibacter* (relative abundances: 8.00% ~ 17.24 and 3.07% ~ 7.10%, respectively), and *Romboutsia* (relative abundances: 9.38% ~ 12.28 and 4.32% ~ 7.09%, respectively) (Figure 3B).

**Figure 4:**
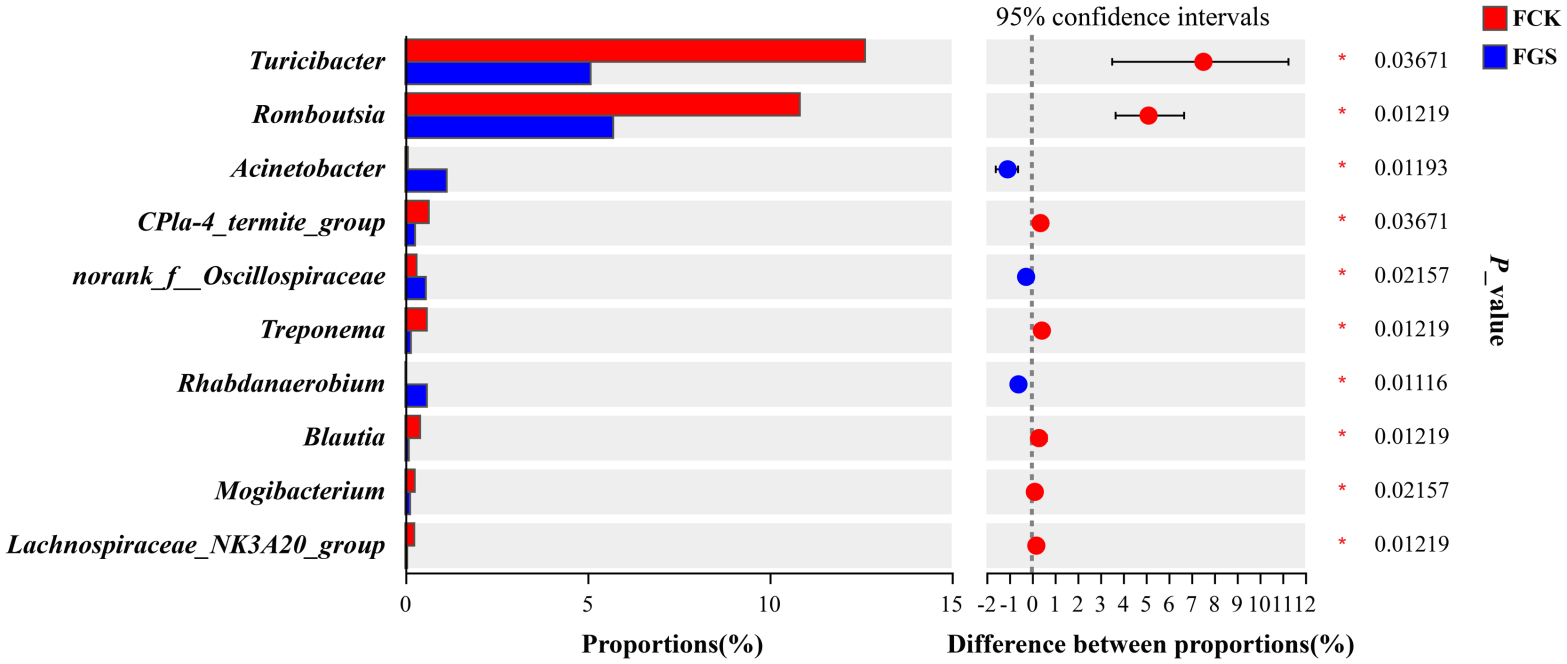
Bar chart for the multi-genera difference analysis. The *y*-axis represents different genera and the colored boxes represent the FCK (red) and FGS (blue) groups. The *x*-axis represents the average relative abundance of a particular genus in the different groups. FCK, fecal bacterial samples collected from the control group; FGS, fecal bacterial samples collected from the *Broussonetia papyrifera* silage treatment group.

Compared with the FCK group, the abundance of *Acinetobacter*, norank_f__Oscillospiraceae, and *Rhabdanaerobium* was significantly higher in the FGS group (*p* < 0.05), whereas that of *Turicibacter*, *Romboutsia*, CPla-4_termite_group, *Treponema*, *Blautia*, *Mogibacterium*, and Lachnospiraceae_NK3A20_group were significantly lower (*p* < 0.05) (Figure 4).

**Figure 5:**
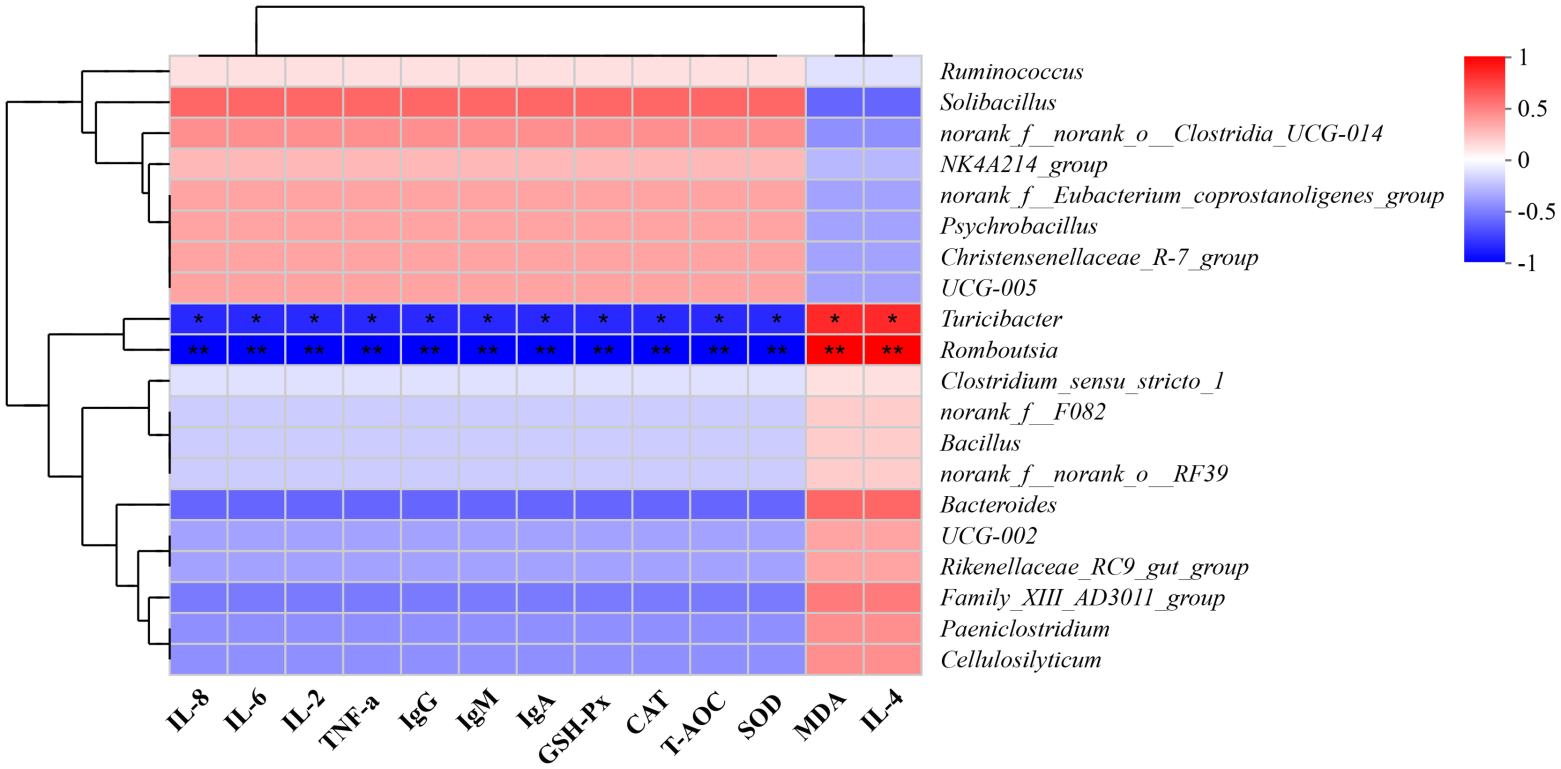
A correlation heatmap of the association of fecal bacteria with antioxidant indexes, immunoglobulin concentrations, and cytokine levels in mucosal tissue of the duodenum, jejunum, and ileum. Antioxidant indexes: T-AOC, total antioxidant capacity; SOD, superoxide dismutase; CAT, catalase; GSH-Px, glutathione peroxidase; MDA, malondialdehyde. Immunoglobulins: IgA, immunoglobulin A; IgG, immunoglobulin G; IgM, immunoglobulin M. Cytokines: TNF-*α*, tumor necrosis factor-alpha; IL, interleukin. The *x*- and *y*-axes represent environmental factors and species, respectively. Correlation coefficients (*R*-values) and *p*-values were calculated. The *R*-values are displayed in different colors on the chart; the color range for the *R*-values is shown on the right of the map. *p ≤ 0.05, **p ≤ 0.01.

As shown in Figure 5, fecal bacteria such as *Turicibacter* and *Romboutsia* exhibited marked positive correlations with TNF-*α*, IL-2, IL-6, IL-8, IgA, IgM, IgG, T-AOC, SOD, CAT, and GSH-Px (*p* < 0.05), while manifesting significant negative correlations with IL-4 and MDA (*p* < 0.05).

## 4 Discussion

### 4.1 Growth performance and apparent digestibility of nutrients

In this study, *B. papyrifera* silage did not affect growth performance or apparent nutrient digestibility in Kazakh sheep. Apparent nutrient digestibility serves as an indicator of both feed nutritional value and the digestive and absorptive capacity of animals. Notably, some studies have suggested that feeding *B. papyrifera* silage can enhance the growth performance of ruminants, potentially due to the resulting variations in dietary nutrient composition (12, 23). However, other research has indicated that feeding *B. papyrifera* silage significantly decreases apparent nutrient digestibility, likely due to the presence of antinutritional factors in *B. papyrifera* that inhibit nutrient absorption (20, 24). Our results differ from these previous reports. We found no significant difference in growth performance between the two groups, which may be attributed to the uniform nutritional level in the diets used in this study. Similarly, no notable differences in apparent nutrient digestibility were observed between the dietary groups, possibly due to the specific lactic acid bacteria preparation used, which may have mitigated the effects of the antinutritional factors associated with *B. papyrifera* silage (4).

### 4.2 Immunoglobulins, antioxidants, and cytokines

Relatively few studies to date have examined the impact of the dietary addition of *B. papyrifera* silage on the intestinal mucosal immune barrier in sheep. The intestine is the largest immune organ in the body and also harbors the highest density of immune cells. In animals, it plays a critical role in mucosal immunity and serves as the first line of defense against infection (25). The levels of immunoglobulins and cytokines can reflect the functionality of the intestinal immune barrier (26). IgA is the primary antibody type in the intestinal mucosa, with IgG and IgM concentrations being present at only relatively low levels. IgA, secreted by intestinal mucosal epithelial cells, plays a protective role during inflammatory responses in the intestinal mucosa (27). Cytokines are key regulators of inflammation and the immune responses triggered by infection or injury (28). T-AOC and SOD, CAT, and GSH-Px contents are essential indicators of the antioxidant capacity of animals, while MDA levels reflect the extent of lipid peroxidation and cellular damage (29–31). The regulatory mechanisms governing cytokine production are complex. The variations in cytokine concentrations observed in this work may be attributed to the multiple antinutritional factors present in *B. papyrifera* silage, which induce intestinal stress in sheep (22). Additionally, some constituents of *B. papyrifera* silage may influence cytokine activity, a possibility that warrants further investigation. The increase in immunoglobulin concentrations (IgA, IgG, and IgM) in the experimental group may not necessarily indicate pathogen exposure but rather reflects the modulation of the intestinal immune system by bioactive compounds present in *B. papyrifera* silage. The dietary incorporation of *B. papyrifera* silage, which contains flavonoids, polyphenolic compounds, and alkaloids, can enhance intestinal health by improving intestinal morphology (16, 32, 33). Although a rise in antioxidant enzymes could suggest mild stress due to bioactive compounds in *B. papyrifera* silage, the overall reduction in MDA levels indicates that these compounds help mitigate oxidative damage, thereby supporting the beneficial antioxidant effects rather than harmful stress. Our results corroborate previous research findings, namely, that the addition of *B. papyrifera* silage in the diet enhances both immunity and antioxidant capacity in animals. Our findings further suggest that such dietary inclusion may bolster the intestinal immune function of sheep and promote overall intestinal health.

### 4.3 Fecal bacteria

Variations in the intestinal bacterial community can reflect the health status and production performance of the host. These communities interact through various signaling pathways, influencing their host’s nutrient metabolism and immune function (34, 35). Several studies have established that the gastrointestinal microbiota of ruminants is predominantly composed of *Firmicutes* and *Bacteroidetes*, in line with the results of this study (36–39). *Firmicutes* primarily facilitate cellulose decomposition, while *Bacteroidetes* enhance carbohydrate utilization by animals (40, 41). In the present study, we noted an increasing trend in the relative abundance of *Firmicutes* in sheep of the GS group, indicative of an improvement in their fiber-decomposing capacity. Conversely, there was a decreasing trend in the abundance of *Bacteroidetes*, suggesting that structural changes within the intestinal bacterial community of the sheep may be linked to antinutritional factors present in *B. papyrifera* silage.

In this work, the relative abundances of *Turicibacter* and *Romboutsia* demonstrated a significant negative correlation with the health of their hosts. *Turicibacter* has been positively associated with inflammation and identified as a target microbe for colitis (42). Additionally, research has shown that mice engaged in treadmill running exhibit a lower fecal abundance of *Turicibacter*, whereas sedentary or forced-exercise mice display the opposite pattern (43). Literature relating to *Romboutsia* remains limited; however, its abundance is significantly elevated in the colonic chyme of rats with irritable bowel syndrome (44). The abundance of *lactobacilli* was increased, whereas that of *Romboutsia* was decreased, in the feces of hens fed diets supplemented with astragalus polysaccharides, indicative of a potential competitive exclusion relationship between these two genera (45). The *B. papyrifera* silage used in this study contained substantial quantities of *lactobacilli*, which may account for the observed decrease in the relative abundance of *Romboutsia*. Notably, the feeding of hybrid mulberry silage led to a significant reduction in the relative abundance of both *Turicibacter* and *Romboutsia*, suggesting that such a dietary intervention can mitigate the prevalence of potentially pathogenic bacterial genera in the host.

## 5 Conclusion

The incorporation of *B. papyrifera* silage in the diet did not impact growth performance or apparent nutrient digestibility in Kazakh sheep. Notably, *B. papyrifera* silage enhanced the immune response and antioxidant capacity of the animals, while concurrently reducing the relative abundance of potentially pathogenic bacteria (*Turicibacter* and *Romboutsia*) in fecal matter. Given the comprehensive bans on antibiotics being introduced within the livestock sector worldwide, *B. papyrifera* silage represents a viable green functional feed for ruminants.

## Data Availability

The datasets presented in this study can be found in online repositories. The names of the repository/repositories and accession number(s) can be found at: https://www.ncbi.nlm.nih.gov/, PRJNA1167466.

## Footnotes

^1^ https://cloud.majorbio.com

^2^ http://www.mothur.org/wiki/Calculators
